# Experimental Infection of Dogs with Toscana Virus and Sandfly Fever Sicilian Virus to Determine Their Potential as Possible Vertebrate Hosts

**DOI:** 10.3390/microorganisms8040596

**Published:** 2020-04-20

**Authors:** Clara Muñoz, Nazli Ayhan, Maria Ortuño, Juana Ortiz, Ernest A. Gould, Carla Maia, Eduardo Berriatua, Remi N. Charrel

**Affiliations:** 1 Departamento de Sanidad Animal, Facultad de Veterinaria, Campus de Excelencia Internacional Regional “Campus Mare Nostrum”, Universidad de Murcia, 30100 Murcia, Spain; clara.munoz1@um.es (C.M.); maria.ortuno2@um.es (M.O.); jortiz@um.es (J.O.); berriatu@um.es (E.B.); 2 Unite des Virus Emergents (UVE: Aix Marseille Univ, IRD 190, INSERM U1207, IHU Mediterranee Infection), 13005 Marseille, France; nazliayhann@gmail.com (N.A.); eagvirology@gmail.com (E.A.G.); 3 EA7310, Laboratoire de Virologie, Université de Corse-Inserm, 20250 Corte, France; 4 Global Health and Tropical Medicine, GHMT, Instituto de Higiene e Medicina Tropical, IHMT, Universidade Nova de Lisboa, UNL, Rua da Junqueira, 100, 1349-008 Lisboa, Portugal; CarlaMaia@ihmt.unl.pt

**Keywords:** host, reservoir, natural cycle, Phenuiviridae, bunyavirales, experimental infection, immunity, neutralizing antibodies, sandfly, *Phlebotomus*, leishmaniasis, meningitis

## Abstract

The sandfly-borne Toscana phlebovirus (TOSV), a close relative of the sandfly fever Sicilian phlebovirus (SFSV), is one of the most common causes of acute meningitis or meningoencephalitis in humans in the Mediterranean Basin. However, most of human phlebovirus infections in endemic areas either are asymptomatic or cause mild influenza-like illness. To date, a vertebrate reservoir for sandfly-borne phleboviruses has not been identified. Dogs are a prime target for blood-feeding phlebotomines and are the primary reservoir of human sandfly-borne *Leishmania infantum*. However, there are no definitive studies to assess whether dogs play a significant role as a reservoir host for human phlebovirus survival in the environment. Here, we have evaluated the susceptibility of domestic dogs to infection by TOSV and SFSV following the direct inoculation of the infectious virus. After experimental infection, the presence of viral RNA was investigated in plasma, urine, saliva, conjunctiva, faeces, semen, and bone marrow samples from 0 to 91 days postinoculation (dpi), as well as in plasma, saliva, and tears samples at 760 dpi. None of the challenged dogs developed clinical signs of infection with either TOSV or SFSV. SFSV RNA was never detected. TOSV RNA was not in any of the specimen types, except for plasma samples that showed low viral loads, although irregularly. None of the dogs developed detectable neutralizing antibodies after a single challenge dose of either TOSV or SFSV. However, a second challenge dose of virus given 56 days later elicited neutralizing antibodies, implying that the first inoculation of virus primed the animals for an anamnestic response following the second challenge. These results demonstrated that healthy domestic dogs are not highly susceptible to infection by TOSV or SFSV and do not develop significant viremia or excrete virus following infection. Consequently, dogs are unlikely natural reservoir hosts of infection and do not appear to play a significant role in phlebovirus transmission cycles.

## 1. Introduction

Sandfly fever is one of the first epidemic arboviral diseases, which have been described [1]. The first and largest epidemic was reported during the Second World War (WWII) in immunologically naïve soldiers from North America stationed in Italy, a phlebovirus-endemic region within the Mediterranean Basin. Infection rates higher than 50% were recorded with soldiers being incapacitated for three days named “3-day fever”. After recognition that the sandfly, *Phlebotomus (P) papatasi*, widely dispersed in peri-Mediterranean countries, was the primary transmission vector of the infectious agents causing the disease, it was also colloquially named “pappataci fever” [1]. Two viruses were isolated from US soldiers, viz., Naples and Sicilian viruses, which are now taxonomically designated as sandfly fever Naples phlebovirus (SFNV) and sandfly fever Sicilian phlebovirus (SFSV), respectively. These viruses are known to be transmitted to vertebrates by sandflies, and since they are antigenically distinct, successive infections in the same individual are not uncommon [2]. Among the genus *Phlebovirus*, Toscana virus (TOSV) and SFSV infect humans and are therefore of public health importance [3]. Although many human infections are mild or asymptomatic, TOSV is also known to be neurotropic and is one of the most common causes of acute meningitis or meningoencephalitis during the warm season in the Mediterranean Basin [4,5,6]. There, sandflies are known to transmit both leishmaniasis parasites and sandfly-borne phleboviruses. Because dogs play a decisive role in the natural cycle of leishmaniasis caused by *Leishmania (L) infantum*, their role as a possible reservoir host for sandfly-borne phleboviruses has been hypothesized [7,8]. Moreover, high phlebovirus seroprevalence rates in dogs have drawn attention to their possible role in the natural virus transmission cycle [8,9,10,11,12,13]. However, there is currently no evidence to suggest that sandflies become infected after biting infected dogs. The duration of viremia in the vertebrate host and consequently the increased likelihood of virus transmission to the vector are good indicators of a potential reservoir ability, as has been observed in other vector-borne viral infections [1]. However, these issues have not been investigated previously for phleboviruses. Arguably, the domestic dog is a good candidate as a reservoir of TOSV and SFSV in the Old World for several reasons: (i) dogs have a close relationship with humans, (ii) they are the primary reservoir of the sandfly-borne zoonotic protozoan, *L. infantum* [14], (iii) they are a frequent blood source for sandflies, and (iv) they develop specific neutralizing antibodies against phleboviruses [5,6,7,8,9,10,11,12,13,15,16]. We experimentally infected dogs with TOSV or SFSV and investigated clinical signs of infection and the presence of virus RNA and/or specific virus antibodies in tissues and body fluids.

## 2. Materials and Methods

### 2.1. Experimental Design, Sampling, and Clinical Examination of Dogs

The study was approved by the University of Murcia Animal Ethics Committee (http://www.um.es/web/vic-investigacion/contenido/vicerrectorado/estructura/comisiones/etica-investigacion) and Murcia´s Regional Government.

**Figure 1:**
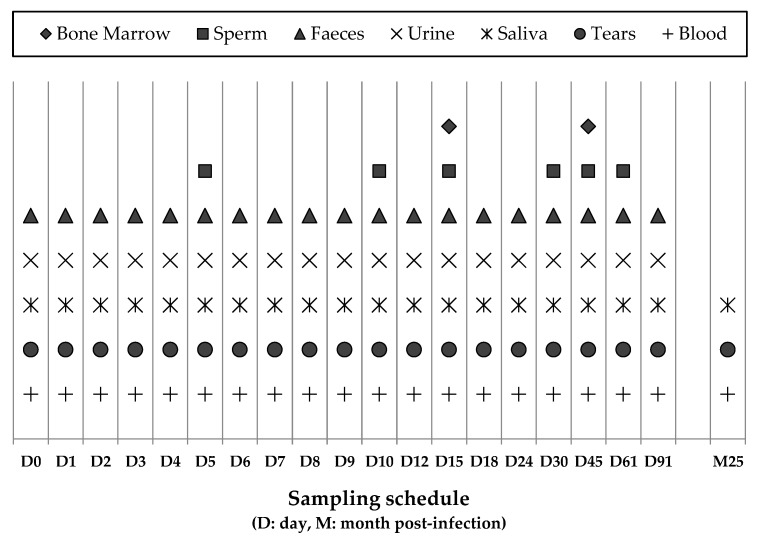
Collection of the specimens from dogs included in the study and the sampling schedule.

The study was carried out in indoor facilities at the University of Murcia (Spain) between November 2016 and February 2017, involved 10 male beagle dogs ranging from 6 to 20 months of age and lasted 91 days. Dogs originated from a commercial breeder in Northern Spain (Isoquimen SL), and prior to the start of the experiment they were vaccinated against rabies, distemper, adenovirus 2, parvovirus, Leptospira interrrogans (Eurican MHPPi2^®^, Boehringer Ingelheim España S.A., Barcelona, Spain), Bordetella bronchiseptica, and Parainfluenza virus (Eurican Bb/PI2^®^, Boehringer Ingelheim) and given a wide spectrum anthelmintic (Prazitel^®^, Ecuphar Veterinaria S.I., Barcelona, Spain). Dogs were provided with an ad libitum, commercial chicken-based pelleted diet (Libra-Adult^®^, Affinity Pet Care, Barcelona, Spain). Four dogs were inoculated intravenously with TOSV (strain 189/ALG/2013), and four were inoculated with SFSV (strain Sabin) obtained from the European Virus Archive collection (https://www.european-virus-archive.com/) (Table 1). Two additional dogs served as uninoculated controls. Viruses were inoculated via the cephalic vein at the start of the experiment (D0) and 56 days post-inoculation (dpi). On D0, two dogs in each virus group received a higher virus dose (10^7^ Tissue-Culture Infectious Dose infecting 50% of cells [TCID_50_]), and the other two received a lower virus dose (10^4^ TCID_50_). At 56 dpi, the dogs in each group received a further 10^7^ TCID_50_ dose of the virus (SFSV or TOSV). The dogs were kept in four pens in the same building with two for each virus, and there was no direct contact between the pens. The uninfected control dogs, inoculated with a saline solution, were each penned with the two dogs infected with the high TOSV and SFSV doses, respectively. The viability of the two viruses inoculated into the dogs was confirmed, using Vero cell cultures, to demonstrate a titratable cytopathic effect, and viral sequences were amplified by PCR as described. The first samples were collected immediately before injecting the virus on D0, and the dogs were then sampled as described in Figure 1. During the experiment, samples of blood, urine, saliva, tears, and faeces were collected on 19 occasions. Semen samples were taken six times, and bone marrow from the costochondral junction was sampled twice. Blood samples were collected in EDTA tubes to obtain plasma, whereas saliva, tears, faeces, and bone marrow samples were collected in tubes containing a viral transportation medium (MW950S, Sigma Virocult^®^, MWE Medical Wire & Equipment, Corsham, Wiltshire, England ). Samples were immediately aliquoted and frozen at −80 °C and analyzed, after the experiment was completed. Before sampling, the dogs were weighed and examined clinically, and their body temperatures were measured. Blood samples were also collected at 0, 1, 3, 7, 15, 30, and 91 dpi for haematological and biochemical tests, including hematocrit (HCT), white blood cell counts (WBCs), lymphocytes, platelets, C-reactive protein (CRP), ferritin, albumin, total proteins, haptoglobin, globulins, creatine kinase (CK), alkaline phosphatase (ALP), gamma-glutamyl transferase (GGT), aspartate aminotransferase (AST), alanine aminotransferase (ALT), creatinine, and blood urea nitrogen (BUN). Moreover, samples of blood, tears, and saliva were collected at 760 dpi from all dogs except for one dog infected with a low SFSV dose.

### 2.2. RT-qPCR Diagnosis

Total nucleic acids were extracted from the collected samples using a QiaCube HT automate with the Cador^®^ Pathogen 96 QIAcube^®^ HT Kit (both from Qiagen) according to the manufacturer’s instructions. Before extraction, the aliquot was spiked with a combination of quantified MS2 and T4 bacteriophages (MS2 and T4) to serve as extraction and inhibition controls. The presence or absence of viral RNA was then investigated with a reverse transcription and quantitative PCR (RT-qPCR), employing a one-step assay combining reverse transcription and PCR amplification in the same reaction tube (EXPRESS One-Step SuperScript^®^ qRT-PCR Kit; Invitrogen, Carlsbad CA, USA). The RT-qPCR used a Taqman probe and primers targeting a fragment of the S-segment of the viral genome for both viruses [17]. RNA from the cultured virus and sterile distilled water were used as positive and negative controls, respectively, for PCR amplification. Thermocycling conditions included the synthesis of complementary DNA at 50 °C for 15 min, followed by denaturation at 94 °C for 2 min, 40 subsequent PCR denaturation at 95 °C for 15 s, and annealing-extension cycles at 60 °C for 1 min. Amplification threshold cycles (Ct) defined as the cycle, in which near logarithmic product generation occurs, were used to quantify the viral RNA load [18]. The Ct was inversely proportional to the number of template copies present in the samples. 

### 2.3. Sensitivity and Limit of Detection (LoD)

The sensitivities of the two selected assays were tested with serial dilutions of TOSV and SFSV of the synthetic RNA prepared using an AVE buffer containing 1 µg/mL of RNA carrier (QIAGEN, Venlo, The Netherlands), to achieve 5-fold serial dilutions. Six decreasing concentrations were tested using six replicates for each. A Ct of ≥40 was considered as negative. The LoD was defined as the number of RNA copies per microliter contained in the highest dilution, for which all 6 replicates were positive.

### 2.4. Neutralizing Antibody Detection

Plasma samples were tested for the presence of neutralizing antibodies against TOSV and SFSV using the virus microneutralization assay, as previously described [19]. The assay was performed in 96-well plates using Vero cells (ATCC CCL81) obtained from a confluent-monolayer culture. Briefly, after preheating plasma at 56 °C for 30 min, 50 μL aliquots of serial plasma dilution were mixed with 50 μL aliquots of infectious virus containing 100 TCID_50_. Plates were incubated at 37 °C with 5% CO_2_ atmosphere for one hour, and 100 μL of Vero cells in suspension containing approximately 2 × 10^5^ cells/mL in 5% fetal bovine serum, 1% Penicillin-Streptomycin, 1% L-Glutamine and 1% Kanamycin-enriched MEM medium (200 mM) were then added to each well, to obtain final plasma dilutions (v/v) ranging from 1:5 to 1:5120. The plates were incubated at 37 °C in an atmosphere containing 5% CO_2_. Positive and negative control wells, containing virus and Vero cells and Vero cells only, respectively, were included in each antibody analysis. After incubation for 5 days for TOSV and 6 days for SFSV, the plates were examined for the presence (no neutralization) or absence (neutralization) of cytopathic effect, using an inverted microscope. Samples recording neutralization at dilutions greater than or equal to 1:40 were considered antibody-positive.

### 2.5. Statistical Methods

The distribution of PCR threshold cycles, neutralizing antibody titers, and biochemical and hematological results were examined, and medians across groups were compared using the nonparametric Kruskal–Wallis test. Differences were considered significant with a *p*-value of <0.05 for a double-sided test. All analyses were performed using the r program (http://cran.r-project.org/).

## 3. Results 

### 3.1. Clinical, Hematological and Biochemical Responses of Dogs Inoculated with SFSV or TOSV

Inoculated and control dogs were monitored daily. None developed clinical signs compatible with either phlebovirus infection or any other infection during the study. Rectal temperatures ranged from 37.8 to 39.4 °C (fever in dogs is defined by temperatures higher than 39.5 °C). All dogs showed moderate weight increases during the experiment (Table 1). 

Results from the biochemical and hematological analyses, according to the experimental group, are shown in Figures S1–S8 in Supplementary Materials. There was no evidence of anemia, thrombocytopenia, or kidney dysfunction judged by normal HCT, platelets, creatinine, and BUN values (Figures S1 and S2 in Supplementary Materials). GGT and AST were normal in all animals (Figure S3 in Supplementary Materials). In contrast, ALP and ALT activity were well above normal levels in two dogs in the TOSV group from D0 before the experimental inoculation until the end of the trial (Figure S4 in Supplementary Materials). Similarly, two dogs in the SFSV group had high WBCs and lymphocyte counts from the start of the trial (Figure S5 in Supplementary Materials). Levels of acute-phase proteins ferritin and CRP were normal except for CRP in one control dog at 1 and 3 dpi and one SFSV dog at 7 dpi (Figure S6 in Supplementary Materials). Moreover, haptoglobin was above normal levels in some dogs in all groups particularly until 30 dpi (Figure S7 in Supplementary Materials). Finally, albumin levels were normal for all dogs, and globulin and total protein concentrations were within or slightly below the normal range in some dogs in all groups (Figures S7 and S8 in Supplementary Materials). 

### 3.2. TOSV and SFSV Detection and Viral Loads

**Figure 2:**
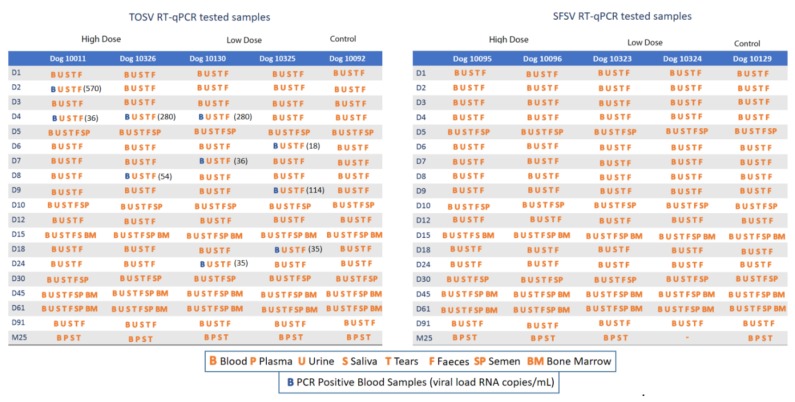
Sequential sampling and viral loads in TOSV- and SFSV-inoculated dogs.

In total, 1035 samples were analyzed. Samples, taken at D0 before dogs were inoculated with TOSV or SFSV, and those from the two uninoculated control dogs were negative. All samples collected from dogs challenged with SFSV were negative for viral RNA, implying either the lack of SFSV replication in dogs (Figure 2) or the failure to detect viral RNA below threshold levels at the time of sampling. On the other hand, TOSV RNA was detected in two to four plasma samples of all four TOSV-inoculated dogs but in none of the other samples (Figure 2). The time and postinoculation for peak viral RNA loads varied in each dog, ranging from 2 to 9 dpi, and the viral load ranged from 18 to 570 RNA copies per milliliter. Four TOSV RNA-positive samples corresponded to two dogs (two samples each, D2/D4 and D4/D8) challenged with high virus doses, whereas six TOSV RNA samples were identified from two dogs (three samples each at 4, 7, and 24 dpi and at 6, 9, and 18 dpi) challenged with low virus doses. Eight of the 10 positive samples were identified between 2 and 9 dpi, and the other two were collected at 18 and 24 dpi (Figure 2), suggesting the possibility that although RNA was detected relatively early after inoculation it was not rapidly cleared from the infected animals. In all cases, RT-qPCR Ct values were high ranging from 31 to 35, and the median (range) Ct was 33 (31–35 and 36–570 RNA copies/mL) and 34.5 (32–35 and 18–280 RNA copies/mL) in positive samples from dogs infected with high and low doses, respectively (*p* = 0.3191). Thus, despite the detection of TOSV RNA in each of the four inoculated dogs, little, if any, viral replication took place. This is emphasized by the fact that TOSV-positive samples were not detected on successive days. Thus, these results do not provide evidence of TOSV replication in dogs inoculated with either low or high doses of TOSV. 

### 3.3. Virus Neutralizing Antibodies in Dogs Inoculated with TOSV or SFSV

**Figure 3:**
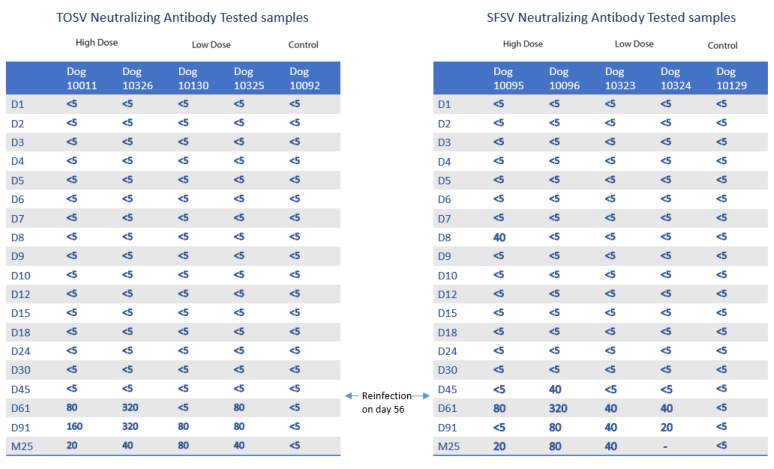
Temporal dynamics of positive neutralizing antibody titers in TOSV- and SFSV-inoculated dogs. M25 = 760 dpi.

The results of all virus neutralization tests on sera taken from TOSV- or SFSV-inoculated dogs are summarized in Figure 3. Overall, 16 samples were antibody-positive, with nine for SFSV and seven for TOSV. However, except for two positive samples (dog 10096 at 45 dpi; dog 10095 at 8 dpi) all the positive sera were detected following the second homologous challenge with high-dose virus at 56 dpi. Both control dogs were antibody-negative for each virus. Until 91 dpi, antibody titers ranged from 1:20 to 1:320 for SFSV and from 1:80 to 1:320 for TOSV. When considering TOSV and SFSV together, median antibody titers were greater in dogs given a high dose of virus (*p* < 0.05), compared with those in dogs given a low dose of virus, and the results of each virus were slightly different from each other (*p* < 0.10). Overall, median antibody titers against TOSV were significantly greater than those against SFSV (*p* < 0.05). Besides, all infected dogs still had neutralizing antibodies at 760 dpi, and titers ranged from 1:20 to 1:80 for both TOSV and SFSV.

## 4. Discussion 

We examined the infection dynamics of TOSV and SFSV in dogs, experimentally inoculated with these viruses. None of the inoculated dogs developed clinical signs of infection, despite the detection of low levels of TOSV RNA in random plasma samples independent of the virus dose. The lack of significant viral RNA replication in plasma samples and the failure to detect excreted viral RNA in saliva, semen, urine, tears, and faeces suggested that healthy dogs were resistant to infection by TOSV and SFSV and therefore probably did not play a significant role as reservoir hosts in TOSV or SFSV transmission cycles. However, specific neutralizing antibodies were detected at 30, 60, and 760 dpi in all experimentally infected dogs, following a second virus challenge administered at 56 dpi. These results are not surprising, since the high virus dose given to each dog was an effective second immunizing dose, and it would therefore be expected to trigger an anamnestic response.

Surveillance for antibodies against TOSV or SFSV in dogs is epidemiologically useful as an indirect estimate of the rate of exposure to the virus and its circulation in a particular region. Canine seroprevalence rates based on virus neutralization tests, the most stringent and specific serological assays for sandfly-borne phleboviruses, show extensive variation across the endemic Mediterranean subregion from 4% in France to 40% in Turkey for TOSV and from 38% in Tunisia to 72% in Greece for SFSV [9,10,12,13]. Although almost 50 percent (48%) of dogs from Southern Spain had TOSV-reactive IgG, using indirect immunofluorescence assays (IFA), these figures must be treated with caution, because cross-reactions among phleboviruses within a given serocomplex are common when using low stringency tests such as IFA, complement fixation, or ELISA [15]. Nevertheless, according to the serosurveillance results quoted here, the high prevalence of neutralizing antibodies against TOSV and SFSV most likely reflected multiple exposures of dogs to these viruses.

The sporadic and irregular detection of TOSV RNA by PCR in plasma but not in other samples from experimentally inoculated dogs, at best, suggested an extremely low and transient level of viral replication. However, the small and irregular quantities of TOSV RNA detected probably reflected the survival of minimal or threshold (barely detectable) levels of input virus. Thus, based on our experiments with Beagles, the likelihood of dogs being a significant reservoir for the maintenance of SFSV and/or TOSV in their natural habitat appeared to be very low. Nevertheless, contrasting results were reported in Turkey, where TOSV RNA was detected in approximately 10% of the tested dogs. Some of the dogs were sick with concomitant leishmaniasis, whereas others were asymptomatic [16]. Co-infection with the *Leishmania* parasite may contribute to this apparent active replication of TOSV. Future studies will be needed to determine whether infection with *Leishmania* can promote the replication of TOSV and/or other related phleboviruses. For example, human volunteers experimentally inoculated intravenously with SFSV were able to transmit infectious virus to *P. papatasi*, which were fed to them at 4 and 5 dpi. Subsequently, hamsters became infected, when these infected sandflies took a bloodmeal from hamsters [20]. This study therefore demonstrated that intravenous administration of SFSV to humans, resulting in infection. This route was previously used with SFSV, and human volunteers developed fever, myalgia, photophobia, and low back and retro-orbital pain [21]. Notwithstanding this, the most natural TOSV infection in humans is subclinical [22]. Likewise, the relatively short duration of viremia and the lack of persistent infection in humans question the role of humans in the maintenance of the virus. Some authors argued that humans are dead-end hosts and do not actively participate in the phlebovirus transmission cycle [23] although they are highly susceptible to TOSV and develop strong assumed lifelong immune response after infection [23]. It has also been considered that sandflies alone may be able to maintain these viruses in the population using venereal and transovarial transmission, along with their ability to survive in diapausing 4th instar larvae during the winter [24]. However, as mentioned before, in the absence of reinfection, these transmission routes may be insufficient to maintain virus endemicity [9,10]. 

TOSV was shown to replicate in endothelial and dendritic cells in vitro and in mice infected subcutaneously [25]. These mice presented TOSV RNA in plasma, and the whole blood samples were taken at 3 and 6 dpi. In another similar experimental study in adult mice, viremia was detected at 6 and 15 dpi, and viral RNA was found in the brain, spleen, and lymph nodes, suggesting their possible involvement in the expansion of the infection [26]. The present study did not include the postmortem examination of organs to detect virus. It is reasonable to believe that the virus would be present in body fluids and/or bone marrow having the virus caused a successful multisystemic infection.

The prerequisites for an animal species to be an efficient reservoir host or amplifying host are as following: (i) generating a high and or sustained viremia upon infection in order to transmit the virus to naïve competent arthropods (sandflies in the current study); (ii) having a geographical distribution that is equal to or higher than that observed for the disease. Our results support the view that healthy dogs are not highly susceptible to infection by either TOSV or SFSV and are therefore unlikely to be a significant reservoir host for these viruses in their natural habitats. 

## 5. Conclusions 

Healthy dogs are not highly susceptible to infection with either TOSV or SFSV and are therefore unlikely to serve as reservoir hosts for these sandfly-transmitted viruses in their natural habitats. 

## Figures and Tables

**Table 1:** Types and doses of virus inoculated into dogs and their ages, body temperatures (°C), and weights (kg) during the experiment.

Dog #	Virus	Infection Dose	Age (Months) at D0	Body Temperature Range (°C)	Body Weight (kg) at the Start of the Experiment (D0)	Body Weight (kg) at 91 Days Postinoculation (dpi)
10011	TOSV ^1^	High ^3^	10	37.8–38.8	14.2	15.2
10326	TOSV	High	20	38.3–39.3	17.5	17.5
10130	TOSV	Low ^4^	10	38.2–38.8	15.8	17.4
10325	TOSV	Low	6	38.1–38.9	17.9	21.5
10092	TOSV	None ^5^	10	38.4–39.4	13.3	16.5
10095	SFSV ^2^	High	10	38.0–39.4	19.3	21.1
10096	SFSV	High	10	38.2–38.9	15.2	17.0
10323	SFSV	Low	7	38.4–39.1	11.3	13.3
10324	SFSV	Low	7	37.8–39.3	11.6	15.1
10129	SFSV	None	10	37.9–38.7	13.6	15.6

^1^, Toscana virus; ^2^, Sandfly fever Sicilian phlebovirus; ^3^, 10^7^ TCID_50_; ^4^, 10^4^ TCID_50_; ^5^ uninfected control.

## Supplementary Materials

The following are available online at https://www.mdpi.com/2076-2607/8/4/596/s1, Figure S1: Hematocrit and platelets values of experimental groups in different experimental days, Figure S2: Creatinine and blood urea nitrogen (BUN) values of experimental groups in different experimental days; Figure S3: gamma-glutamyl transferase (GGT) and aspartate aminotransferase (AST) values of experimental groups in different experimental days; Figure S4: Alkaline phosphatase (ALP) and alanine aminotransferase (ALT) values of experimental groups in different experimental days; Figure S5: White blood counts (WBCs) and lymphocytes values of experimental groups in different experimental days; Figure S6: Ferritin and C-reactive protein (CRP) values of experimental groups in different experimental days; Figure S7: Albumin and haptoglobin values of experimental groups in different experimental days; Figure S8: Globulin and total protein values of experimental groups in different experimental days.
