# Supplementary material for: Experimental Infection of Dogs with Toscana Virus and Sandfly Fever Sicilian Virus to Determine Their Potential as Possible Vertebrate Hosts
Source: Microorganisms. 2020 Apr 20;8(4):596. doi: 10.3390/microorganisms8040596 (PMC7232252; doi:10.3390/microorganisms8040596)
Supplement: Supplementary file 1 [file microorganisms-08-00596-s001.pdf]

Supplementary Figures

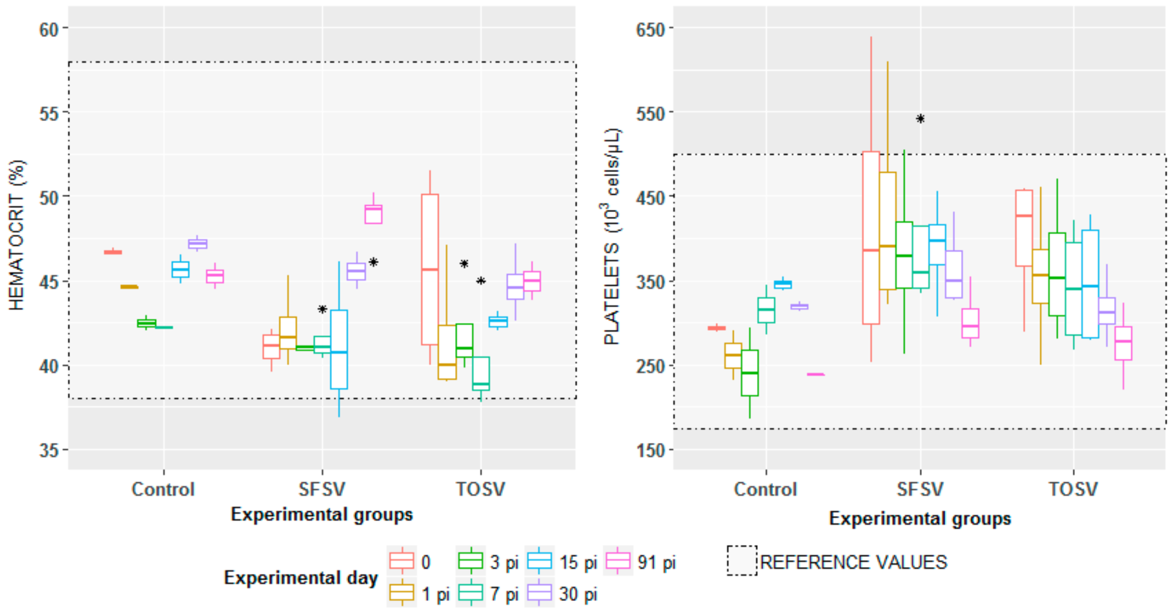

Supplementary Figure 1. Hematocrit and platelets values of experimental groups in different experimental days

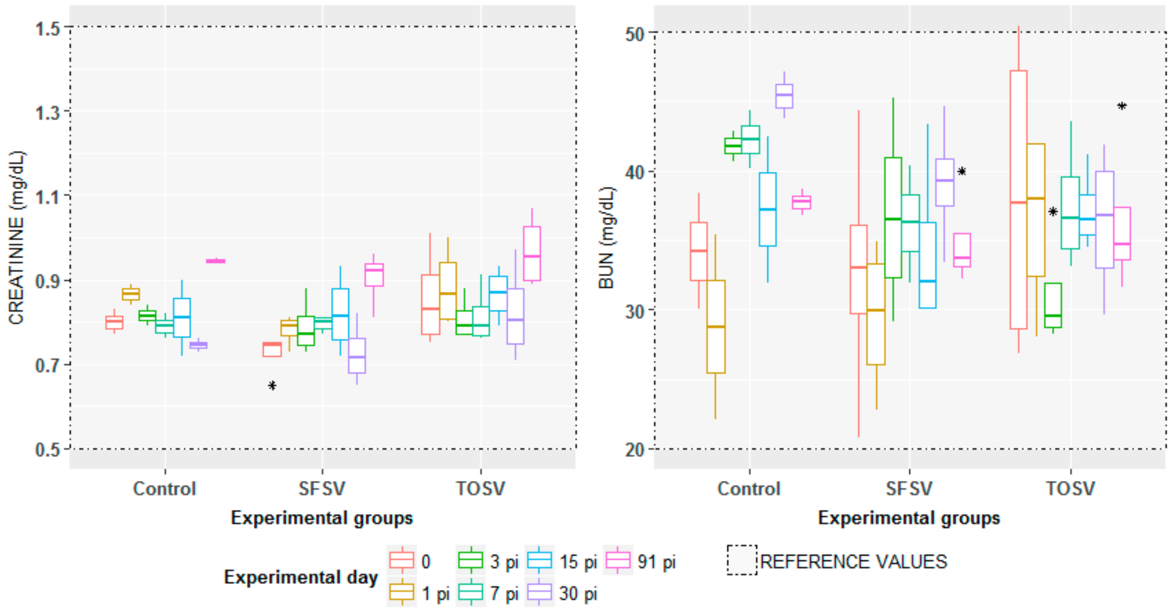

Supplementary Figure 2. Creatinine and BUN values of experimental groups in different experimental days.

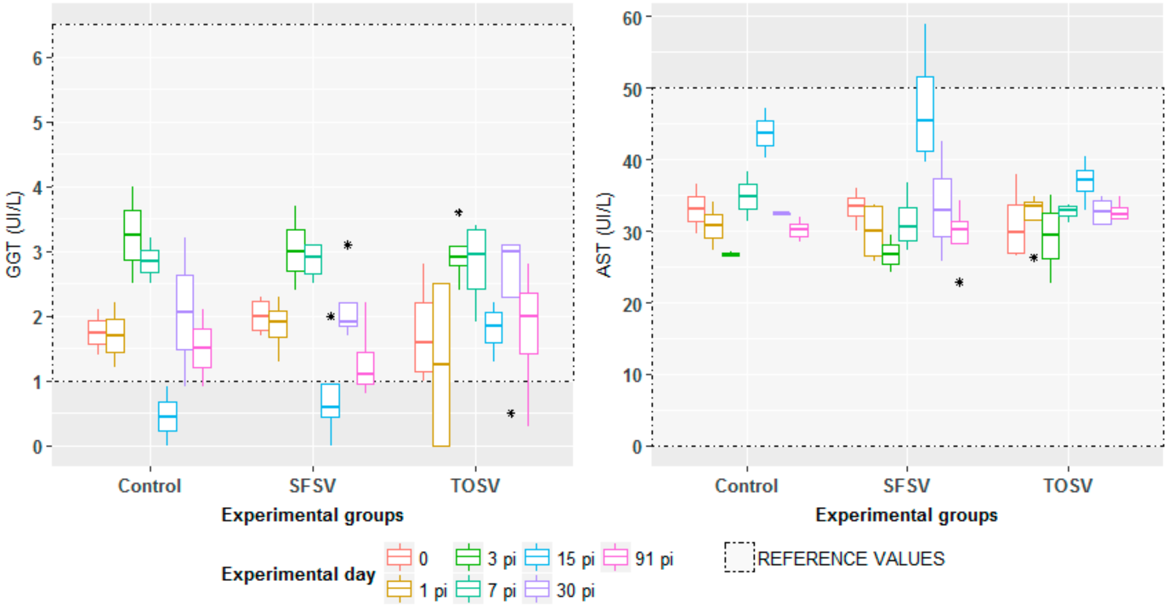

Supplementary Figure 3. GGT and AST values of experimental groups in different experimental days.

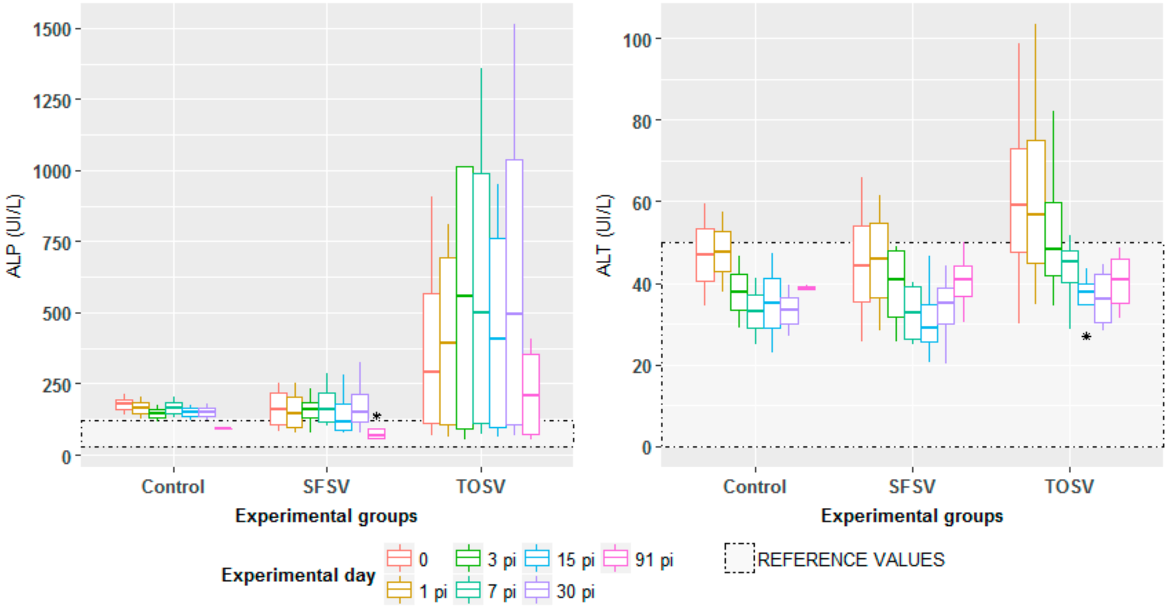

Supplementary Figure 4. ALP and ALT values of experimental groups in different experimental days.

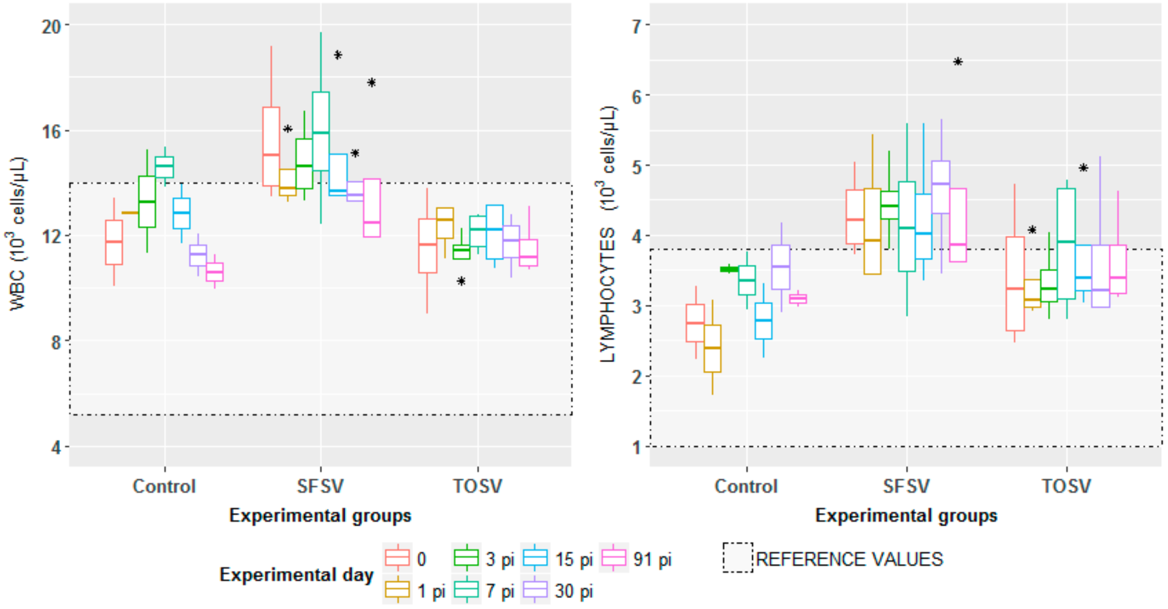

Supplementary Figure 5. White blood cells (WBC) and Lymphocytes values of experimental groups in different experimental days.

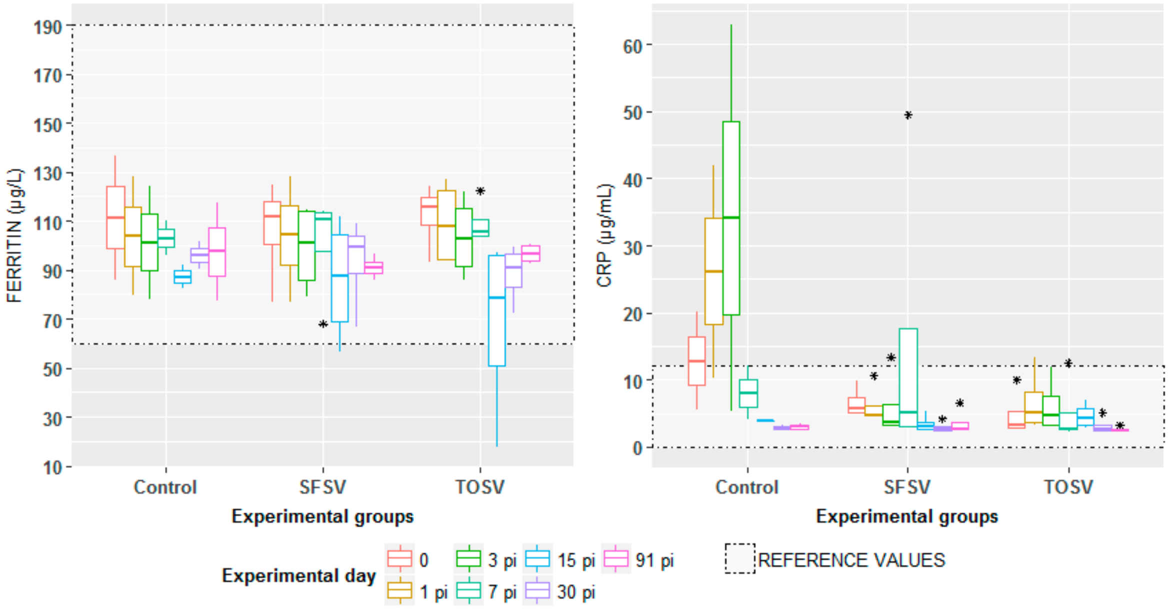

Supplementary Figure 6. Ferritin and CRP values of experimental groups in different experimental days.

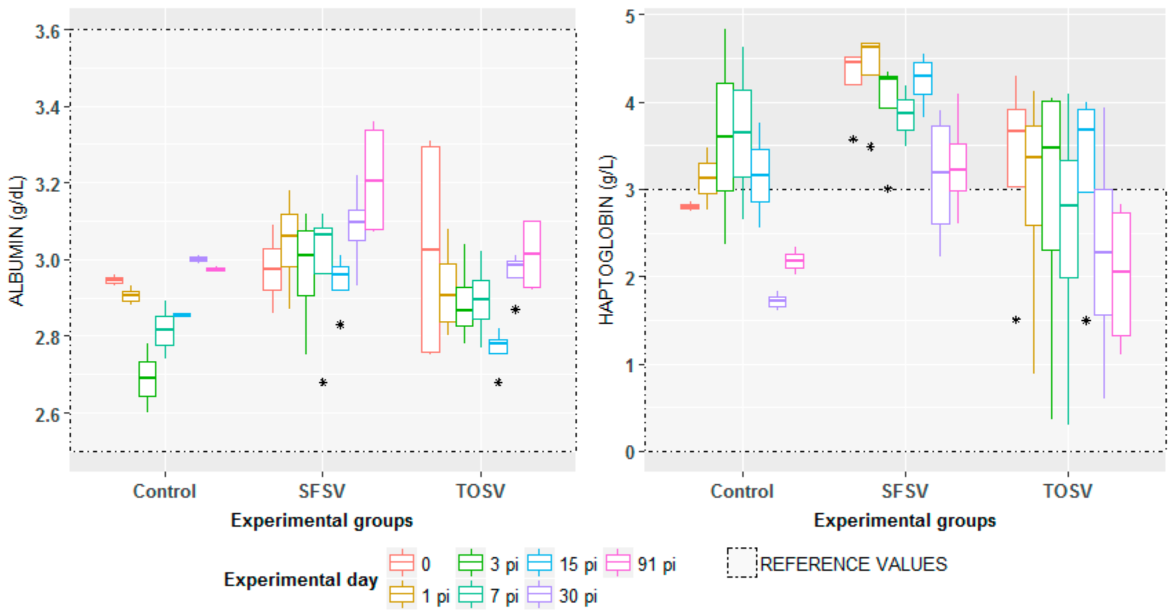

Supplementary Figure 7. Albumin and Haptoglobin values of experimental groups in different experimental days.

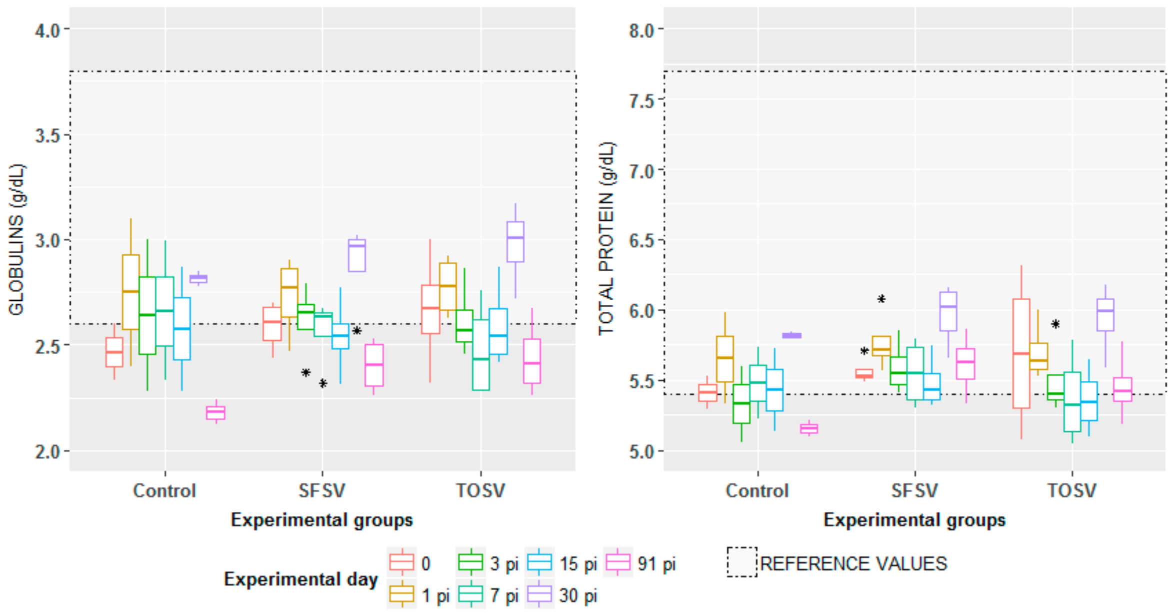

Supplementary Figure 8. Globulin and Total protein values of experimental groups in different experimental days.
